# Asylum Dysentery

**Published:** 1901-04-20

**Authors:** 


### ASYLUM DYSENTERY.

According to a report presented to the London County Council by Dr. Mott and Dr. Durham, on colitis or asylum dysentery, the signs of this disease are in many cases not well marked, so that beyond certain abnormal characters of the evacuations it is difficult to distinguish it from simple diarrhœa. The mainstay of the diagnosis in fact depends upon the observation of blood and slime in the stool. The most interesting part of the report is that which is devoted to an attempt to elucidate the etiology of the disease, tending as it does to show the necessity, wherever large numbers of people are gathered together, of taking the greatest pains to prevent the occurrence of any short circuit between the food supplies and the arrangements made for the removal and utilisation of excreta. Although it may be admitted that the infection on which the disease depends might be conveyed by air or water, there does not seem to have been any proof that such was the case in the outbreaks which were investigated. But under the heading defective sanitation attention is directed to several matters which evidently demand speedy rectification, even though there may be no proof that they were the causes of the mischief. Take, for example, the manner in which an outflow of raw, unscreened, untreated sewage at Claybury is made to irrigate beds of lettuce and celery which are intended for eating in an uncooked condition. It seems that this sewage matter when examined was not only swarming with bacteria, but contained abundant unaltered fæces, so that we need not for a moment hesitate to condemn such a use of such a fertiliser, even although it may be found impossible to give proof positive that this crude method of sewage disposal was the cause of the dysentery which prevailed among the inmates of this asylum. Various other matters are pointed out which all indicate a lack of intelligent supervision, and a readiness to become the slave of ill-contrived regulations and red tape. Will it be believed that in some cases it was found to be the custom to lock up the ward lavatories after the morning wash until the next morning, so that even where patients might have to assist in attending to diarrhœa cases the only way by which they could clean their hands was by washing at the slop sink, at which, as occurred in one instance in the presence of Dr. Mott and Dr. Durham, the excreta of a patient suffering from dysentery were disposed of. Overcrowding, again, and a system of packing the patients together in their dormitories which would hardly be credible were it not stated in black and white in an official report, are doubtless responsible for much, but they would hardly produce dysentery were they not combined with the extremely defective arrangements for securing personal cleanliness which seem to prevail in these great asylums. Many of the things mentioned in this report can only be described by the word disgraceful, and the calm acceptance of them by all concerned as if they were inevitable is perhaps one of the most miserable parts of this miserable *exposé*. There can hardly be a doubt that asylum dysentery is a filth disease which is caused by the filthy conditions which have apparently become traditional in these asylums, notwithstanding all the "inspection" to which they are subjected. Inspection indeed!

